# The control of *Hyalomma* ticks, vectors of the Crimean–Congo hemorrhagic fever virus: Where are we now and where are we going?

**DOI:** 10.1371/journal.pntd.0010846

**Published:** 2022-11-17

**Authors:** Sarah I. Bonnet, Gwenaël Vourc’h, Alice Raffetin, Alessandra Falchi, Julie Figoni, Johanna Fite, Thierry Hoch, Sara Moutailler, Elsa Quillery

**Affiliations:** 1 Animal Health Department, INRAE, Nouzilly, France; 2 Ecology and Emergence of Arthropod-borne Pathogens Unit, Institut Pasteur, CNRS UMR 2000, Université Paris-cité, Paris, France; 3 Université Clermont Auvergne, INRAE, VetAgro Sup, UMR EPIA, Saint-Genès-Champanelle, France; 4 Université de Lyon, INRAE, VetAgro Sup, UMR EPIA, Marcy l’Etoile, France; 5 Reference Centre for Tick-Borne Diseases, Paris and Northern Region, Department of Infectious Diseases, General Hospital of Villeneuve-Saint-Georges, 40 allée de la Source, Villeneuve-Saint-Georges, France; 6 EA 7380 Dynamyc, UPEC, Créteil, France; 7 Unité de recherche EpiMAI, USC ANSES, Ecole Nationale Vétérinaire d’Alfort, Maisons-Alfort, France; 8 UR7310, Faculté de Sciences, Campus Grimaldi, Université de Corse, Corte, France; 9 Santé publique France, 94410 Saint-Maurice, France; 10 French Agency for Food, Environmental and Occupational Health & Safety, 14 rue Pierre et Marie Curie, Maisons-Alfort Cedex, France; 11 Oniris, INRAE, BIOEPAR, Nantes, France; 12 ANSES, INRAE, Ecole Nationale Vétérinaire d’Alfort, UMR BIPAR, Laboratoire de Santé Animale, Maisons-Alfort, France; Beijing Children’s Hospital Capital Medical University, CHINA

## Abstract

At a time of major global, societal, and environmental changes, the shifting distribution of pathogen vectors represents a real danger in certain regions of the world as generating opportunities for emergency. For example, the recent arrival of the *Hyalomma marginatum* ticks in southern France and the concurrent appearance of cases of Crimean–Congo hemorrhagic fever (CCHF)—a disease vectored by this tick species—in neighboring Spain raises many concerns about the associated risks for the European continent. This context has created an urgent need for effective methods for control, surveillance, and risk assessment for ticks and tick-borne diseases with a particular concern regarding *Hyalomma* sp. Here, we then review the current body of knowledge on different methods of tick control—including chemical, biological, genetical, immunological, and ecological methods—and the latest developments in the field, with a focus on those that have been tested against ticks from the genus *Hyalomma*. In the absence of a fully and unique efficient approach, we demonstrated that integrated pest management combining several approaches adapted to the local context and species is currently the best strategy for tick control together with a rational use of acaricide. Continued efforts are needed to develop and implement new and innovative methods of tick control.

## Author summary

Disease-bearing *Hyalomma* ticks are an increasingly emerging threat to humans and livestock worldwide. Various chemical, biological, genetic, and ecological methods for tick control have been developed, with variable efficiencies. Today, the best tick control strategy involves an integrated pest management approach.

## Introduction

Ticks have a worldwide distribution, occupy a wide variety of biotopes and comprise about 900 species, some of them being able to transmit viruses, bacteria, and/or parasites [1]. In the Northern Hemisphere, they are the primary vectors of both human and animal pathogens, and in the Southern Hemisphere, they are the primary vectors of importance in animal health [2]. Socioeconomic and climate trends drive changes in the geographic distribution and seasonality of several tick species, their vertebrate hosts, and the reservoirs of pathogens, shaping the persistence of pathogen foci since the beginning of the 20th century [3–8]. Thus, the question of the consequences of the introduction and/or permanent establishment of new tick species with their pathogens in areas previously devoid of them is now center stage. Ticks of the genus *Hyalomma* represent a perfect example associated with this risk of emergence [9]. In fact, *Hyalomma marginatum* is regularly introduced—but to date unable to establish itself—via migratory birds in Northern Europe [10–12]. However, the recent establishment of this species in the South of France [13], along with the occurrence of Crimean–Congo hemorrhagic fever (CCHF) cases in recent years in Spain [14], raises many concerns about the associated risks for the European continent.

**Fig 1:**
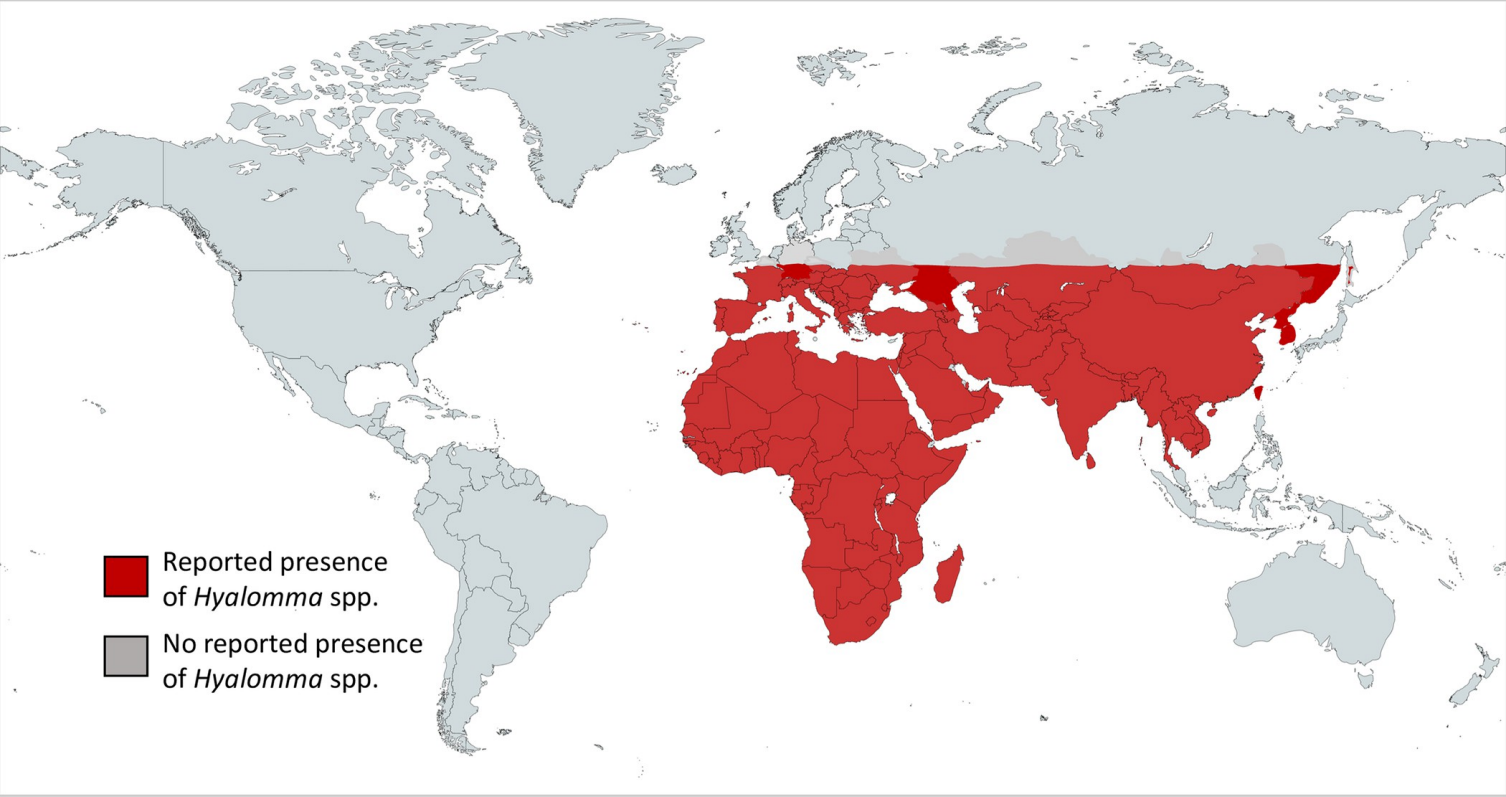
Global geographic distribution of *Hyalomma* spp. ticks. Created with mapchart.net.

**Fig 2:**
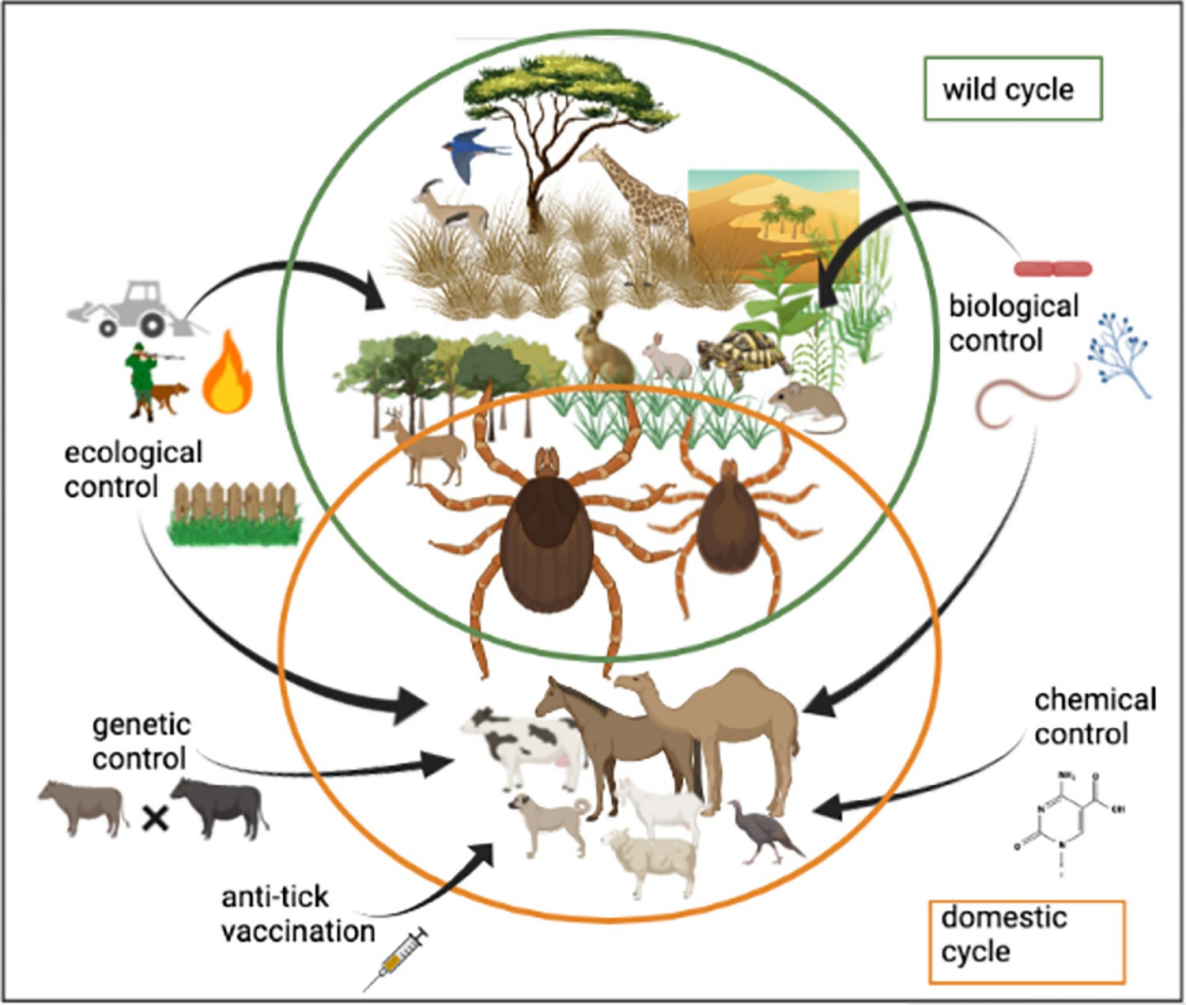
Schematic representation of the different control methods applicable to *Hyalomma* ticks for consideration in the context of integrated pest management. Created with BioRender.com.

Currently, 27 species are recognized within the genus *Hyalomma* and are present on all continents except in North and South America [15]. Their distribution is conditioned by their preference for open spaces with relatively hot and dry climates (semidesert steppes, savannas, Mediterranean scrubland, etc.) (Fig 1). Like all hard ticks, *Hyalomma* have 3 developmental stages: larvae, nymphs, and adults (males and females), each of which takes only 1 blood meal. The majority of *Hyalomma* species have a triphasic cycle, with larvae and nymphs mostly choosing small vertebrates in sheltered habitats as hosts, whereas adults mainly feed on large ungulates including livestock (Fig 2). The *Hyalomma* species most frequently infesting humans are *Hy*. *anatolicum*, *Hy*. *marginatum*, and *Hy*. *aegyptium* [16,17]. *Hyalomma* spp. adults have generally an ambush behavior towards their host. They have low self-dispersal, but can nonetheless be transported over long distances by their hosts during their blood meal [18].

A large number of pathogens—parasitic, viral, or bacterial—have been mentioned in the scientific literature as being transmitted or potentially transmitted by ticks from the genus *Hyalomma*; all have animal reservoirs and some cause zoonoses [19]. Nevertheless, very few vector-competence experiments have been conducted due to the difficulties in carrying out a complete cycle under experimental conditions (the need for “healthy” tick colonies, for vertebrate hosts adapted to both ticks and pathogens or for an efficient artificial tick feeding technique, for pathogen cultures, as well as for high biosafety level facilities, etc.). Among the viruses for which transmission by some *Hyalomma* species has been proven are the CCHF virus [20], the Dugbe virus [21], the West Nile virus [22], the Venezuelan equine encephalitis virus [23], the African horse sickness virus [24], and the Kyasanur Forest disease virus [25]. Among bacteria, some *Hyalomma* species are involved in the transmission of *Rickettsia aeschlimannii* [26], *Anaplasma marginale* [27], and *Coxiella burnetii* [28,29]. Finally, *Hyalomma* species can transmit to cattle *Theileria annulata*, the agent of tropical theileriosis (TT), or Mediterranean theileriosis, *Theileria orientalis*, the agent of oriental theileriosis [30], and *Babesia occultans* [26,31]. In small ruminants, they transmit *Theileria lestoquardi* and *Theileria ovis* [32,33], and in equids *Theileria equi* and *Babesia caballi* [34,35]. Apart from pathogen transmission and in addition to significant blood spoliation, *Hyalomma* ticks also generate serious wounds that can become superinfected during the bite due to their long mouthparts [36,37].

The evidence for the medical and veterinary importance of *Hyalomma* species continues to accumulate over time. As with all other arthropod vectors, effective control of these ticks can only be achieved with a good understanding of the biology and ecology of the species in their natural habitat. In the context of increasing resistance to acaricides, integrated control of these arthropods is to be favored. Integrated control is based on the rational application of a combination of chemical, physical, biological, genetic, ecological, or animal population selection measures to keep the presence of vectors below a certain threshold of pathogen transmission or economic loss. Here, we explore the current body of knowledge on the various tick control methods and the latest developments in the field, with a special focus on the results obtained for *Hyalomma* ticks.

### Chemical control

The use of acaricides in tick control dates back to the late 19th century, with the use of baths containing arsenic derivatives to eliminate *Rhipicephalus microplus* [38], and remains the main method of tick control used today. Acaricides are still applied directly to the vulnerable host subject to tick infestation, either by spraying or bathing individuals with aqueous acaricide formulations, or by topical treatments on the back (with a “pour-on” formulation). Exceptionally, in the case of massive infestation by endophilic ticks on a farm, the application of acaricides to the walls of stables may be recommended [39]. However, in the case of *Hy*. *scupense*, for example, this technique is only marginally effective because nymphs can penetrate even deep cracks and crevices [39]. Due to the appearance of tick populations resistant to certain insecticides and their strong negative impact on the environment, arsenic derivatives, organochlorines, and then organophosphates were abandoned in the 1970s in favor of pyrethroids (e.g., deltamethrin, permethrin, flumethrin), which inactivate sodium channels [40], neurotoxins such as fipronil and amitraz [41], growth inhibitors such as fluazuron [42], or activators of invertebrate glutamate-dependent chloride channels such as macrocyclic lactones (e.g., eprinomectin, ivermectin, moxidectin) [43].

To date, resistance to all of these substances has been identified in ticks, making their effectiveness increasingly uncertain [44]. Although most of the studies carried out on tick population control using chemical acaricides, or resistance to them, involve species of *Rhipicephalus* (*Boophilus*)—monotropic ticks that infest cattle in many regions of the world and thus subject to very strong selection pressure [44]—, a few studies have focused on *Hyalomma* sp. suggesting that resistance in certain populations is emerging, especially in Asia. In Pakistan, tests conducted in 2009 did not show resistance to ivermectin or cypermethrin in *H*. *anatolicum* [45], but resistance to fipronil and ivermectin was identified in 2021 [46]. These 2 studies had unsurprisingly identified both a misuse of acaricides over exceedingly long periods of time (often coupled with low concentrations or inadequate doses) and lack of product rotation as risk factors for the development of resistance. In India, other populations of *H*. *anatolicum* have been identified as resistant to deltamethrin and diazinon as well as fipronil and cypermethrin [47–49]. Resistance to emamectin benzoate, spirotetramat, and hexaflumuron has also been reported in populations of *H*. *asiaticum* in China [50]. In contrast, a 1996 study showed the efficacy of flumethrin as a pour-on for camels against *H*. *dromedarii* [51]. The efficacy of an amitraz/cypermethrin pour-on mixture was also validated for *H*. *rufipes* infesting buffaloes in South Africa in 2005 [52].

In addition to the development of acaricide resistance, the negative impacts of acaricides include contamination of livestock production (milk and meat) and the environment, including non-target animals [53]. For this reason, research is being carried out to identify new acaricide substances that are more “natural” and/or less damaging to the environment, such as substances derived from plants, essential oils, or nanoparticles. Validating their efficacy and formulations adapted for administration to animals is, however, time consuming and still requires research [54–56]. A recent comprehensive list of plant-derived substances tested against *Hyalomma* species is presented in a recent review [57]. A 2017 review also presents the efficacy levels of metal and metal oxide nanoparticles tested as acaricides against ticks with a high level of toxicity to *H*. *anatolicum* and *H*. *isaaci* larvae [58]. The value of using tick hormones has also been evaluated in other species and has been shown to increase the efficacy of acaricides after inclusion in “a permethrin-impregnated oily matrix” [59], or coupled with a “tail-tag decoys” in cattle [60,61]. In addition, traps using these pheromones coupled with or without CO_2_ have also been developed with some success against *Amblyomma* spp. [62,63]. However, the trials carried out have not led to the marketing of these traps.

### Biological control

Ticks have a number of natural enemies (bacteria, fungi, spiders, ants, beetles, hymenopterans, rodents, birds, etc.), and several studies have investigated control methods that can take advantage of their action.

Among the entomopathogenic fungi evaluated for activity against ticks, *Beauveria bassiana*, *Lecanicillium (Verticiliium) lecanii*, and *Metarhizium anisopliae* have been the most studied [64]. They have been commercially developed as biopesticides. Results show that the pathogenicity of the fungus depends on the tick species, tick life stage, tick feeding intensity, the fungus strain, and also the weather conditions and the season in which the conidia are deployed [64]. For example, very encouraging results have been obtained, both in vitro and in vivo by ground spraying with solutions containing *M*. *anisopliae* conidia, either against *R*. *microplus (*reviewed in [65]), or against *Ixodes scapularis* [66]. A few trials have been also conducted with entomopathogenic nematodes [67], or with bacteria such as *Bacillus thuringiensis* [68].

Experiments have also been carried out with a tick parasitoid hymenopteran, *Ixodiphagus hookeri*. In the first half of the 20th century, attempts to control ticks by releasing *I*. *hookeri* were carried out in the United States against *Dermacentor* ticks and in the Soviet Union against *Ixodes ricinus* ticks [69]. Though initially effective, these releases did not prevent the tick population from maintaining itself, probably due to the much higher fertility of ticks compared to the parasitoid wasps [69]. More recently, a trial was conducted in Kenya where 150,000 parasitoids were released in 1 year on a 4-ha pasture occupied by 10 tick-infested cattle [70]. The results showed an impact on *Amblyomma variegatum* ticks, with 50% of the ticks being parasitized by *I*. *hookeri*, and a reduction in the tick infestation of cattle, but a parallel increase in the *Rhipicephalus appendiculatus* tick population. Above all, it appeared that it was necessary to release parasitoid wasps frequently to obtain significant effectiveness. Moreover, for *I*. *ricinus* ticks and under natural conditions, there is a balance between the ticks and the parasitoids, the latter infecting only a small proportion of the ticks and having no effect on the global population [71].

Among vertebrate predators, few are specialized in eliminating ticks, with the exception of oxpecker birds (*Buphagus erythrorhynchus* and *B*. *africanus)*. A study performed in South Africa on birds in captivity showed that they can eat up to 100 engorged females of *Rhipicephalus decoloratus* or more than 7,000 larvae of *Amblyomma hebraeum* per day [72]. However, the use of these birds for effective biological control is unlikely to be feasible. In Africa, it has also been observed that chickens can be effective in controlling ticks, which they consume in large numbers [73,74].

Other invertebrate predators with a significant impact on ticks are species of *Lycosa* (wolf spiders) and especially ants. For example, in some areas of Australia favorable to *R*. *microplus* ticks, the absence of ticks has been attributed to the predatory action of ants [75]. Petney and colleagues [76] suggest that ant predation on free ticks is higher than generally reported and may even be a factor determining population size. Ants of the genus *Solenopsis* have also been observed feeding on engorged females of *Rhipicephalus* (*Boophilus*) or *Amblyomma* [76–79]. However, the predatory activity of ants is extremely variable and unpredictable in the field and precise data are scarce [75,80].

With specific regard to *Hyalomma*, trials have been carried out with fungi, nematodes, and entomopathogenic bacteria. A commercial formulation of the *Beauvaria bassiana* fungal species was sprayed in rabbit holes to control *H*. *lusitanicum* in Spain. The quantity of ticks collected from rabbits 30 days after treatment was reduced by almost 80% in spring, but by only 36% in summer, pointing to the importance of temperature on the effectiveness of biocontrol based on fungi [81]. When tested in vitro on engorged *H*. *anatolicum* females, this fungus also showed a synergistic effect when used with deltamethrin [82]. When used alone, *B*. *bassiana*, like *M*. *anisopliae*, induces 90% mortality in these same ticks [82]. The in vitro efficacy of *M*. *anisopliae* against all stages of *H*. *anatolicum* collected in Sudan was then confirmed [83], as well as that of *Scopulariopsis brevicaulis* against adult tick stages [84]. Finally, a study demonstrated, in vitro, an effective ovicidal effect of proteases produced by *Aspergillus sojae* and *A*. *oryzae* on *H*. *dromedarii* eggs [85]. Biological control trials on *Hyalomma* ticks using entomopathogenic nematodes have also been carried out in laboratory conditions, and high virulence of different strains of *Steinernema* sp. and *Heterorhabditis* sp. has been reported against *H*. *dromedarii* and *H*. *excavatum* [86–88]. Finally, *B*. *thuringiensis* can act on the hemocytes of *H*. *dromedarii*, suggesting a possible toxic action of this bacterium against this tick species [89].

Although currently, in addition to their high costs, none of these methods taken alone have proven to have sufficient effectiveness on a large scale, some have shown encouraging results, making it possible to consider their use in the context of integrated pest management [90]. Nevertheless, in addition to questions of stability of the results obtained over time and the method of application, before implementing them on a large scale in the field, it will be necessary to ensure that non-target arthropod fauna is not affected.

### Genetic control

Genetic control of arthropod vectors consists in altering/modifying their reproductive potential or other functions of interest to combat the vectors or the pathogens they transmit via modifications to their genetic make-up. The genetically modified vectors are then released into the wild to compete with natural populations. The success of such a control strategy therefore depends, among other things, on the existence of a selective advantage of the replacement population. Here, under the term “genetic control,” we also include the selection of animal breeds that are naturally more resistant to tick infestation.

Despite recent progress in tick genetic manipulation [91], the possibilities for implementing genetic tick control strategies are very limited. Firstly, breeding large numbers of ticks in the laboratory, an integral part of this strategy, is not an easy task [92]. In addition, the low dispersal capacity of ticks in the natural environment—even though they can travel long distances via their hosts during their blood meal—and their biology, including very long development cycles, represent an obstacle to the replacement of natural populations by genetically modified ones [18]. Finally, in addition to the lack of data on tick genomes, which hinders the identification of potential targets for genetic control, the release of genetically modified organisms into the wild currently raises numerous questions (ethical, legal, logistical, etc.). However, as early as 1976, laboratory irradiation trials had shown the effectiveness of sterilizing *H*. *anatolicum* males [93]. Irradiation doses sufficient for the production of sterile males that still compete with untreated males for mating with females, thus limiting female fertility, were then established for *H*. *anatolicum* [94,95], *H*. *marginatum* [96], and *H*. *excavatum* [97]. Sterilization of males is also possible in *Dermacentor variabilis* by blocking *subolesin* expression through RNA interference [98]. Nevertheless, to our knowledge, no field release trials of sterile males have been carried out to date for ticks.

The existence of tick-resistant cattle breeds was observed as early as 1912 in Australia in a Jersey herd infested with *R*. *microplus*: Some animals were always less infested than others, and the trait appeared to be hereditary (see review by Burrow and colleagues [99]. Since then, a number of studies have demonstrated the existence of breeds that are more or less resistant to ticks, with variable heritability of the trait. For example, *Bos indicus* (zebu) is more resistant to infestation by *R*. *microplus* or *Amblyomma americanum* than *Bos taurus* (cattle) [100,101]. One study identified 3 key pathways in this phenomenon, represented by genes differentially expressed in resistant individuals: the development of the cell-mediated immune response, structural integrity of the dermis, and intracellular Ca^2+^ levels [102]. Another study suggests the involvement of the interferon-gamma pathway [103]. In the 1970s, several cattle breeds resistant to *R*. *microplus* were thus created, selected, and bred in Australia: Australian Friesian Sahiwal [104], Australian Milking Zebu [105], and Australian Illawarra Shorthorn [106]. However, they were met with mixed success, with farmers preferring the use of acaricides against which ticks had not, at the time, developed much resistance [107]. Work on the Belmont Adaptaur cattle breed shows that tick resistance can be increased to very high levels via selection [108]. More recently, a cross-breeding trial between sires and dams with low *A*. *variegatum* infestation was conducted in Goudali zebu cattle in Cameroon, but without conclusive results [109]. Resistance is reflected in the rejection of ticks (especially larvae), a lower gorging rate and thus lower weight in females despite an increased attachment time, and a lower hatching rate. In the 1990s, breeding resistant cattle was presented as a promising approach to control tick populations [110], but this has not yet led to applications usable by farmers and no significant progress has been made on the subject since 2000 [111].

The majority of studies have since focused on *R*. *microplus*, a single-host tick species [112]. However, both innate and acquired resistance to ticks has also been observed with 2- or 3-host ticks, such as *Amblyomma* spp. [100,113–115] or *Ixodes* spp. [116], but also *Hyalomma* spp. Studies have shown acquired resistance to *H*. *anatolicum* bites in rabbits [117–119] and in crossbred (*B*. *indicus* X *B*. *taurus*) calves [120,121]. In South Africa, reports indicate that indigenous Nguni cattle are less infested with *Hyalomma* sp. than Bonsmara or Hereford cattle [115] and that Brahman cattle are less infested with *H*. *rufipes* than Simmental cattle [122]. Studies in the Gambia have also shown better resistance to *Hyalomma* sp. infestation in N’dama (*B*. *taurus*) cattle than in the Gobra (*B*. *indicus*) breed [123]. In Ethiopia, the local Arssi breed was found to be the most resistant to *H*. *rufipes* bites, followed by the Boran breed, and Boran x Friesian crossbred cattle were the least resistant [124]. Finally, a study in Morocco also showed that a local breed was more resistant to *H*. *marginatum*, *H*. *detritum*, *H*. *anatolicum*, and *H*. *lusitanicum* than Friesian cattle [125].

### Anti-tick vaccination

The evidence of natural immunity to tick bites, identified as early as the beginning of the 20th century (see recent reviews: [111,126]), and capable of providing indirect protection against tick-associated diseases, has naturally led the scientific community to consider anti-tick vaccine control strategies. By targeting the vector, anti-tick vaccines have the dual advantage of combating both the ticks themselves and the direct losses they cause, as well as all potentially transmitted pathogens [127]. Two main types of anti-tick vaccines have been developed, those using antigens exposed to host immune responses during gorging, such as salivary antigens, and those that rely on canceled molecules not exposed to the host immune system [128]. When a tick feeds on a vaccinated animal, it will ingest the antibodies induced by the vaccination, which will diffuse throughout the tick body and inhibit the targeted functions: digestive functions in the digestive tract, anticoagulant, immunosuppressive or anti-inflammatory functions in the salivary glands, or those involved in the multiplication and/or transmission of pathogens. As a result, the tick may be prevented from feeding or digesting, may be rejected by the host immune system, and/or there will be no transmission of microorganisms to the host [129–133]. However, the development of this anti-tick vaccination strategy faces many challenges including the lack of genomic data on ticks, the difficulty of obtaining experimental models, the cost of development and competition with the acaricide industry, as well as the selection of the vertebrate hosts that need to be vaccinated, particularly for multi-host species [134].

Thus, despite extensive research on the subject, only 1 vaccine against *R*. *microplus* is currently marketed in Cuba as Gavac and used in many Central and South American countries [135,136]. In controlled field trials in Cuba, Brazil, Argentina, and Mexico, the Gavac vaccine shows 55% to 100% efficacy in controlling *R*. *microplus* infestations in grazing cattle 12 to 36 weeks after the first vaccination [137]. A similar vaccine was previously marketed in Australia, where it was originally developed, under the name TickGARD [136]. These vaccines are based on a tick digestive tract antigen (Bm86) and generate an immune response that interferes with the digestion of the blood meal and thus decreasing the tick population, the number of eggs laid by females being directly related to the volume of ingested blood [138]. Although this vaccine provides proof of feasibility for anti-tick vaccination, it has the disadvantage of being effective almost exclusively against *R*. *microplus*, with notable variations in efficacy depending on tick populations and regions of the world, and of being expensive, particularly due to the booster shots required [139].

Could this vaccine be used against *Hyalomma*? Several studies have been conducted, with contrasting results. Vaccination with Bm86 showed no effect on infestations of cattle with adult *H*. *scupense* or *H*. *excavatum* ticks [139]. In contrast, an earlier study showed that vaccination of cattle with Bm86 resulted in a 30% reduction in the number of engorged *H*. *anatolicum* nymphs, and even a 95% reduction in the number of feeding nymphs for *H*. *dromedarii* [140]. A similar result was subsequently reported with 89% reduction in the number of feeding nymphs for *H*. *dromedarii* in vaccinated cattle, but the vaccine was less effective in camels, with a reduction in feeding nymph numbers of only 27% [141]. Furthermore, this type of vaccine will be more effective for monotropic species such as *R*. *microplus* that feed almost exclusively on 1 animal species and have a rapid cycle with several generations per year [129]. For broad host-spectrum species with a single annual generation, vaccination must eliminate the tick as soon as it attaches or prevent the transmission of pathogens in the vaccinated animals [132]. Vaccination can even target wild animals: A recent vaccination trial in deer reduced infestation with different tick species, including *H*. *marginatum* and *H*. *lusitanicum* [142].

However, research has turned to the identification and use of Bm86 orthologs in other tick species, including some belonging to the *Hyalomma* genus, because the ineffectiveness of the Bm86-based vaccine against various *Hyalomma* species (including *H*. *excavatum* and *H*. *marginatum*) has been attributed to the large sequence variations identified in the homologous proteins of these species [143]. Vaccination against Haa86, a homolog of Bm86 in *H*. *anatolicum*, has shown a protection rate of 60% to 82% depending on the study and has been shown to reduce transmission of *T*. *annulata* in cattle [144–146]. Vaccination of cattle against Hd86, a Bm86 homolog in *H*. *scupense*, showed a 59% reduction in the number of engorged nymphs, but no impact on adults [147–149]. More recently, ATAQ, a protein paralogous to Bm86, has been identified in the gut and Malpighian tubules of all Metastriata group of hard ticks (i.e., *Rhipicephalus*, *Amblyomma*, *Hyalomma*, *Dermacentor*, *Haemaphysalys*, and *Bothriocroton* genera) [150]. Although no vaccination trials have yet been carried out, ATAQ appears to be a promising vaccine candidate due to its homology with Bm86 and its conservation across several genera.

The majority of studies investigating the possibility of using other molecules than Bm86 or its orthologs as vaccine candidates to protect against *Hyalomma* sp. infestation have focused on *H*. *anatolicum*. Immunization with extracts of this tick has shown a decrease in tick feeding in several studies, in some cases with a decrease in transmission of *T*. *annulata* [151–158]. A subolesin homolog that has shown some efficacy against *R*. *microplus* has been identified in *H*. *anatolicum*, but there have been no vaccine trials to date [159]. In another study, subolesin, calreticulin (CRT), and cathepsin type L (CathL) show some reduction of feeding capacity of *H*. *anatolicum*, on the order of 65%, 41%, and 30%, respectively [160]. In cattle, immunization with ferritin-2 (FER2) and tropomyosin (TPM) has recently shown protection rates against *H*. *anatolicum* larvae and adults between 51% and 66% [161]. Finally, vaccination trials of rabbits with glycoproteins extracted from *H*. *dromedarii* showed a slight decrease in the reproductive index of engorged females and a significant reduction in egg-hatching rates [162].

With the exception of Bm86 (for certain tick populations), no tick antigen has thus far been shown to be sufficiently effective in protecting against tick infestation or pathogen transmission. It is likely that the implementation of effective vaccination would require the use of several antigens responsible for partial blockage of feeding and/or pathogen transmission in a “cocktail” vaccine [163]. Thus, very recently, in silico studies have led to the construction of an as-yet untested vaccine candidate that would include both structural protein epitopes of the CCHF virus and subolesin as an antigen against ticks in order to offer dual protection against CCHF transmission [164].

### Ecological control

Ecological control, which consists of creating unfavorable conditions in the environment for the ticks to complete their cycle, mainly targets the environment and the tick–host populations [69,165,166]. Another mode of action is to act directly on the probability of encounter between ticks and their hosts [167]. Most of the available literature on these methods involve family *Ixodidae* ticks, but the same principles can be considered for *Hyalomma*.

Environmental modification can have a direct role in affecting tick activity and survival. For example, clearing low vegetation has been shown to be effective in reducing populations of *I*. *ricinus* [168] or *A*. *americanum* [169]. Fire can also have a direct effect on tick survival as well as an indirect effect via the impact on host populations and vegetation, and annual burns constitute an effective method of tick control [170]. For example, temporary reductions in tick populations have been observed for *I*. *scapularis* after fires [171]. But the challenges and risks associated with this practice mean that the use of fire to control tick populations should be considered with great caution, especially because fires have also been associated with increases in *A*. *americanum* (presumably because deer were attracted to the renewed vegetation growth) [172] or *R*. *appendiculatus* [173], or with no effect on *Ixodes pacificus* in California [173]. It has also been suggested that the use of plants that are repellent or toxic to ticks could limit their populations [69]. Experimentally, Civitello and colleagues [174] showed that Japanese stiltgrass (*Microstegium vimineum*) increases the mortality of *A*. *americanum* and *D*. *variabilis*. However, this strategy seems difficult to implement over large areas and would require these species to be highly dominant [175]. In addition, environmental modification can have an indirect role on tick populations by affecting host abundance and diversity, and thus the probability of contact between a tick and a host.

Most ticks have trophic preferences, attaching and feeding more easily and efficiently on certain animal species. Thus, the control of these host species, mainly through hunting or exclusion with barriers, can have an impact on tick populations. Gilbert and colleagues [176] have shown in Scotland that *I*. *ricinus* tick populations are smaller in forests or heathlands where hunting pressure is higher, and that, 4 to 5 years after the fencing of experimental sites, tick populations are less abundant. More dramatically, Rand and colleagues [177] showed that the removal of white-tailed deer (*Odocoileus virginianus*) from an island on the East Coast of the United States—where no other wild species is known to potentially host adult ticks—resulted in an increase in questing adult *I*. *scapularis* in the year following eradication of their preferred host, but a drastic drop in all stages in later years. However, the effect of reducing host populations in areas where host movements are not controlled is more nuanced [178]. For example, controlling *I*. *ricinus* tick populations by excluding deer appears to be effective in protecting the human population in specific areas [179], but excluding white-tailed deer to limit the abundance of *A*. *americanum* varies in effectiveness across different years [180].

Regarding *Hyalomma*, a study in Spain comparing an open and a closed area from which deer and wild boar had been excluded for 16 years showed that *H*. *lusitanicum* populations were more abundant in the open areas [181]. In a theoretical study based on a population dynamics model, it was shown that the decrease in density of hares, which host the immature stages of *H*. *marginatum*, is associated with a decrease in adult tick populations after 5 years of simulation [182]. Furthermore, studies have shown that under certain conditions in environments with relatively low host biodiversity, tick abundances in the environment are higher compared with environments harboring higher biodiversity. In environments with high biodiversity, the probability of encounter between ticks and their preferred host is reduced and feeding success is lower due to grooming behavior or resistance to ticks of some of the hosts present. For example, in the United States, *I*. *scapularis* feeds on a wide variety of hosts [183]. In addition to the species richness of environments, functional diversity is also important, in particular, the presence of predators that may limit host populations or limit the foraging time of these hosts (and thus potential contact with exophilic ticks) [184,185]. To our knowledge, no such studies have been conducted on *Hyalomma* ticks.

For tick species that feed on livestock, rotational grazing may reduce host infestation by removing animals from a pasture plot for the duration necessary for the free-living ticks present there to die out by starvation [75,186]. This practice is particularly suitable if animals are treated with an acaricide at each change of pasture plot, thus avoiding immediate reinfestation of the pasture by the engorged ticks introduced by the animal host. However, this strategy can only be effective for species in which the survival time of the free-living stages in the environment is not very long, such as for the larvae of *R*. *microplus*, a monophasic tick. This strategy is not possible with 2- or 3-host ticks such as *Hyalomma* spp. whose unfed adults can survive on pasture lands for several years under good conditions [187]. For the rotation system to work, there must also be no alternative hosts in the environment that allow the cycle to be completed in the absence of the domestic host. In some cases, this would require a hermetic fence around the pastures, which is very rarely possible.

For ticks feeding on domestic animals, it is also possible to adapt herd management to the tick activity cycle, but data are lacking for *Hyalomma* spp. Knowledge of their rhythm of detachment at repletion could, for example, theoretically make it possible to favor detachment in places that are unsuitable for their survival or oviposition. For example, as engorged *R*. *microplus* females detach mainly in the morning, holding animals in the morning, before grazing, in night pens or stables where the detached engorged females will not survive, can thus limit the infestation of the environment [188]. In contrast, as engorged *A*. *variegatum* nymphs detach from the host in the afternoon (2 PM to 5:30 PM), if the herds are kept during this period on pastures unfavorable to tick survival, or on plots subsequently cultivated, it can greatly reduce adult tick infestation in the following months [189]. Similarly, noting that *A*. *variegatum* adults are more active during the day than at night, Barré (1998) suggested to encourage the nocturnal grazing of cattle. However, these actions are limited by their feasibility in terms of herd management as well as by the plasticity of tick behavior that can adapt to their environment [190].

Finally, manual tick removal of domestic animal hosts is also used by farmers in smallholdings [191]. This operation mainly targets adult ticks, because larvae and nymphs are difficult to detect, and can secondarily reduce tick densities in pastures. However, the risk of infection must be controlled when handling ticks.

The difficulty with all these control methods lies, among other things, in understanding and taking into account their short- and long-term ecological consequences. On the one hand, the effects observed on the short term are not necessarily those observed on the long term and, on the other hand, the ecological consequences on the ecosystem as a whole are difficult to predict due to the evolution of community dynamics.

### Other avenues of research

Recent advances in the description and study of the tick microbiome (see review [192]) open up new avenues of research to identify strategies for tick control. For example, studies of mosquito vectors have shown that modulating their microbiome reduces the transmission of pathogens responsible for malaria, dengue, and other mosquito-associated diseases [193]. Using their own microbiome as a “weapon” against ticks can be seen from 2 different angles. One approach is to target microbes that are necessary for tick survival or development, such as those responsible for nutrient supplementation or stress reduction; vaccination against *I*. *ricinus* is based on these tick microbes [194]. Another tack is to target components of the tick microbiome that are directly involved in pathogen transmission, by activating or suppressing the immune system, competition for limited resources or regulation of a specific physiological process. One example is the introduction of a vertically transmitted endosymbiont capable of inhibiting pathogen transmission into a laboratory tick population for release into the field to overtake natural populations. Additionally, based on the positive associations between the microbiome and certain tick-borne pathogens, vaccination strategies against these microbiome components may merit development. Clearly, it is now essential to study and understand the functional consequences of interactions between ticks, their microbiome, and pathogens, as in the study of the transmission of *B*. *burgdorferi* or *A*. *phagocytophilum* in *I*. *scapularis* to identify potential vaccine targets [194,195].

Although attractive, this approach to manipulating the microbiome will face a number of challenges that need to be addressed, in addition to the lack of functional data at present. Firstly, the dynamics of the tick microbiome vary not only between species, but also between geographical origin, sex, stage, or blood meal origin [192]. Therefore, these factors will have to be taken into account because they can represent important obstacles to the identification of a common usable target. Secondly, the release of ticks with a genetically modified microbiome into the field will come up against the same difficulties as those mentioned for genetic tick control methods (ethical and regulatory problems, low dispersion, long generation times, etc.). Thirdly, as mentioned above with regard to anti-tick vaccines, it should also be kept in mind that a vaccine approach directed against elements of the microbiome will be complex to implement for multi-host ticks. Compared with other genera, relatively little work has been done on the *Hyalomma* microbiome [196–199]. However, a recent study has demonstrated, using tick samples collected in the field in Pakistan, that populations of *Francisella*-like endosymbionts and *Candidatus* Midichloria mitochondrii, which predominate in female *H*. *anatolicum*, are not altered by the presence of 2 pathogens they transmit: *Theileria* sp. and *A*. *marginale* [200].

Finally, new approaches based on existing, but more environmentally friendly strategies can also be considered in tick control. One example is the very recent development of a pheromone trap which, after attracting *R*. *sanguineus* ticks, kills them by electrocution [201]. This type of trap, which to our knowledge has never been tested on *Hyalomma* sp., could prove effective due to the hunting behavior of adults. The identification of new tick-specific targets, such as substances in their nervous system, may also allow the development of vaccines [202] or new acaricides that preserve non-target fauna [203], particularly in association with nanoparticles [58].

## Conclusion

As with other tick species and genera, there is currently no “miracle solution” for controlling *Hyalomma* spp. According to the danger they represent, whether in terms of transmission of the CCHF virus to humans or of parasites such as *Theileria* sp. to animals, it is now urgent to develop new methods of control that are environmentally sustainable against these very important disease vectors, especially considering current global changes and the growing resistance to acaricides. In that context, an attractive approach for preventing tick-borne diseases can consist of development of vaccines that target conserved tick molecules as it may provide broad protection against current and future tick-borne pathogens. However, it must be kept in mind that these methods must be adapted not only to the biology and ecology of the species involved, for which much data are still lacking, but also to the realities of the field, taking into account the feasibility of their application, as well as the expectations and acceptability of stakeholders and civil society. It is only with the compliance of the relevant populations and within the framework of an integrated control plan aimed at changing practices (breeding, human activities in risk areas, etc.) that solutions to fight ticks, including *Hyalomma* spp., can be implemented.

**Key Learning Points**

- *Hyalomma* spp. ticks are responsible of negative impact both directly due to their bite because they are some ectoparasites and indirectly as important disease vectors for humans and animals.
- The geographic distribution of *Hyalomma* ticks is currently expanding due to global changes, suggesting the possibility of emerging diseases related to these vectors.
- In the absence of vaccine and specific treatment against Crimean hemorrhagic fever (CCHF) virus transmitted by *Hyalomma* sp., the only control method available at present is the control of tick vectors.
- Integrated pest management combining several approaches adapted to the local context and species is currently the best strategy for tick control together with a rational use of acaricide.
- Continued efforts are needed to develop and implement new and innovative methods of tick control.

**Top Five Papers**

1. Gray JS, Dautel H, Estrada-Pena A, Kahl O, Lindgren E. Effects of climate change on ticks and tick-borne diseases in europe. Interdisciplinary perspectives on infectious diseases. 2009;2009:593232.
2. Vial L, Stachurski F, Leblond A, Huber K, Vourc’h G, et al. Strong evidence for the presence of the tick Hyalomma marginatum Koch, 1844 in southern continental France. Ticks Tick Borne Dis. 2016;7:1162–1167.
3. Negredo A, Sanchez-Ledesma M, Llorente F, Perez-Olmeda M, Belhassen-Garcia M, et al. Retrospective Identification of Early Autochthonous Case of Crimean-Congo Hemorrhagic Fever, Spain, 2013. Emerg Infect Dis. 2021;27:1754–1756.
4. Bakheit M, Latif A, Vatansever Z, Seitzer U, Ahmed J. The Huge Risks Due to *Hyalomma* Ticks. In: Mehlhorn H, editor. Arthropods as vectors of emerging diseases. New York: Springer; 2012. p. 167–194.
5. Graf JF, Gogolewski R, Leach-Bing N, Sabatini GA, Molento MB, et al. Tick control: an industry point of view. Parasitology. 2004;(129 Suppl): S427-442.
